# Transdiagnostic patient experiences of dialectical behavioural therapy: a systematic review and metasynthesis

**DOI:** 10.3389/fpsyt.2025.1640341

**Published:** 2025-10-06

**Authors:** Abigail Hall, Lynsey Gregg, Brian O’Ceallaigh, Anja Wittkowski

**Affiliations:** ^1^ Division of Psychology and Mental Health, School of Health Sciences, Faculty of Biology, Medicine and Health, The University of Manchester, Manchester, United Kingdom; ^2^ Perinatal Mental Health and Parenting (PRIME) Research Unit, Research and Innovation Department, Greater Manchester Mental Health National Health Service (NHS) Foundation Trust, Manchester, United Kingdom; ^3^ Greater Manchester Mental Health NHS Foundation Trust, Perinatal Specialist Service, Manchester, United Kingdom

**Keywords:** qualitative research, psychological therapy, patient-centred care, literature review, thematic synthesis

## Abstract

**Background:**

Dialectical Behaviour Therapy (DBT) combines cognitive-behavioural techniques and mindfulness practices to more skilfully regulate intense emotions and navigate interpersonal issues. While traditional DBT (skills group, individual therapy and crisis support) is well-studied in clinical populations, particularly for emotion regulation in conditions like borderline personality disorder or emotionally unstable personality disorder, recent research has explored alternative formats, such as skills-only groups. Although quantitative studies report positive outcomes (e.g., reduced self-injury and suicidality), less is known about patient experiences, which are crucial for developing effective interventions. This systematic review explored patient experiences of DBT skills groups across mental health conditions and age groups, considering the processes patients perceived as contributing to therapeutic change and outcomes.

**Method:**

A systematic search was conducted across five databases following PRISMA guidelines, using search terms related to DBT and patient experience. Peer-reviewed papers employing qualitative or mixed-methods were included. Thematic synthesis was used for analysis.

**Results:**

Thirty-two papers were eligible for inclusion. Three main themes were generated: *1) the challenging road to DBT, 2) the difficult journey through DBT*, and *3) patients’ path for the future.* Theme two contained three sub-themes (*from theory to practice, transformative relationships - self and others, scaffolding and supporting change*) and theme three included two sub-themes (*therapeutic gains, future directions*).

**Conclusions:**

Findings highlight the importance of pre-treatment and in-treatment experiences alongside relational factors like safety and validation and practical skill application. Key processes, including peer support and changed perspectives, shape therapeutic outcomes. Recommendations include flexible delivery formats and aligning patient preferences with intervention to maximise gains.

**Systematic review registration:**

https://www.crd.york.ac.uk/PROSPERO/login, identifier PROSPERO (CRD42024604496).

## 1 Introduction

DBT is a well-established psychological intervention, originally developed by Linehan (1, 2) as a comprehensive treatment for individuals with borderline personality disorder (BPD), which is characterised by interpersonal relationship difficulties and ineffective emotion regulation, often leading to self-injury or suicidality. Originally, DBT was designed as an intensive year-long programme, covering four modules (mindfulness, interpersonal effectiveness, distress tolerance and emotion regulation), to address the chronic and pervasive nature of difficulties faced by those diagnosed with BPD. DBT combined group skills training, individual psychotherapy, telephone coaching and therapist team consultations. Over the past two decades, continued research has demonstrated DBT’s effectiveness in reducing self-injury and suicidal behaviours (3–6).

While DBT research has historically focussed on adults with BPD, its emphasis on addressing emotion regulation difficulties (such as intense mood swings, impulsivity, and interpersonal challenges) has led to its broader application in treating other presentations underpinned by these difficulties, including eating disorders (7), attention-deficit hyperactivity disorder (8) and trauma-related conditions (9–11). Numerous systematic reviews and meta-analyses highlight DBT’s effectiveness across mental health conditions and clinical settings, consistently demonstrating reductions in distress-related behaviours, such as suicidality and self-injury in adults (12–16), and adolescents (17). However, while various studies and reviews provide evidence for DBT’s effectiveness transdiagnostically, they reveal little about the *mechanisms of change* or the *patient experience*.

A growing body of literature emphasises the importance of understanding patient perspectives, including treatment acceptability, satisfaction, and perceived therapeutic gains (18, 19). McPherson et al. (18) argue that while RCTs and meta-analyses are invaluable for assessing efficacy and effectiveness, they often overlook critical contextual factors that shape treatment engagement and outcomes. Without qualitative insights, systematic reviews risk presenting an incomplete understanding of why DBT is helpful for various mental health conditions, and *how* it impacts patients’ daily lives.

Given the resource demands of traditional DBT, a stand-alone DBT skills group format emerged over time through clinical and research adaptations. This format is increasingly used as a cost-effective alternative to traditional DBT (5, 20) given the time and financial constraints healthcare settings often face. For example, DBT skills groups alone have been found to significantly reduce emotional dysregulation and depressive symptoms (21–24). By retaining DBT’s modular structure (mindfulness, interpersonal effectiveness, distress tolerance and emotion regulation) and core principles (such as dialectics, validation, and behavioural change), the stand-alone skills group format offers a more accessible and resource-efficient alternative to traditional DBT programmes for individuals with emotion regulation difficulties across diverse clinical populations. Furthermore, a recent systematic review and meta-analysis of 11 studies with adolescents (25) underscored the therapeutic value and positive impact of these skills groups on suicidal ideation and risk management.

As both stand-alone DBT skills groups and those delivered as part of comprehensive DBT programmes are increasingly used in clinical practice, understanding patient experiences of the group-based components becomes especially important. To date, only two systematic reviews synthesised qualitative or mixed-methods research on patient experiences of traditional DBT. Using a meta-ethnographic approach, Little et al. (26) analysed seven studies and identified four main themes: *life before DBT, supportive relationships for change, shifts in perspectiv*e and the *development of self-efficacy*. More recently, Middlehurst et al. (27) expanded on this work, synthesising 11 studies and highlighting three main themes: *impact of DBT, supportive structure* and *1:1 therapy component.* Advocating for further research to deepen understanding of patient perspectives, the authors of both reviews concluded that adult patient experiences of DBT were complex and multifaceted. Although quantitative research supports DBT’s transdiagnostic effectiveness across different age groups (e.g., adolescents and adults), qualitative insights remain underexplored. The aforementioned reviews focussed solely on adults with a diagnosis of BPD, thereby leaving a crucial gap in the literature regarding patient experiences across ages and mental health conditions.

Thus, in this systematic review we aimed to explore patient experiences of DBT skills groups, whether delivered as stand-alone interventions or embedded within full DBT programmes, across mental health conditions and age groups (adolescents and adults). Additionally, we considered the processes patients perceived as contributing to therapeutic change and outcomes. By addressing this gap, the review could provide valuable insights to support patient-centred care and enhance the refinement of DBT interventions in diverse clinical settings.

## 2 Methods

A systematic review and meta-synthesis, informed by the Preferred Reporting Items for Systematic reviews and Meta-Analyses (PRISMA) guidelines (28) was undertaken to address the review’s aims. The protocol was registered with PROSPERO on 23/10/2024 (Ref: CRD42024604496). The Enhancing Transparency in Reporting the Synthesis of Qualitative Research (ENTREQ) checklist (29) was completed to ensure appropriate reporting of review findings (see **Appendix 1** for completed checklist).

### 2.1 Search strategy

The search strategy was developed in collaboration with the University of Manchester library service, following the Population, Intervention, Comparison, Outcome, and Study Design (PICOS) framework (30) (see **Table 1** for details). A preliminary search indicated that terms associated with the intervention and the study design only were required to achieve appropriate sensitivity and specificity. Specific search criteria did not include “qualitative” because the study design related terms already identified qualitative research. Furthermore, the authors wished to include mixed-method papers which met the eligibility criteria.

**Table 1:** Search terms and search strategy.

Search strategy	PICOS	Search terms
1	P- Population	N/A
2	I- Intervention	Dialectical Behaviour Therapy OR Dialectical Behavioural Therapy OR Dialectical Behaviour Therapy OR Dialectical Behavior Therapy OR Dialectical Behavioral Therapy OR DBT
3	C- Comparison	N/A
4	O- Outcome	N/A
5	S- Study Design	Experience* OR View* OR Attitude* OR Perception* OR Belief* OR Feelings* OR Perspective* OR Opinion*
6	Combine 2 and 5	

Five electronic databases were searched in October 2024, with updated searches completed in February 2025, for peer reviewed articles: CINAHL Plus (EBSCOhost), Web of Science (Clarivate), PsycINFO, EMBASE and Medline (all OVID). Databases were selected due to their relevance to this topic area. Searches were conducted using the terms outlined in **Table 1**, with the use of Boolean operators (“and”, “or”) and were not restricted by publication date. Reference lists of identified papers were also searched.

References were exported into EndNote and duplicates were removed. Title and abstract screening was carried out by the first author, who reviewed papers to confirm they employed qualitative or mixed-methods designs. An independent reviewer carried out a second screening on a random sample of 25% of the identified references. Perfect agreement regarding which papers to include/exclude was found between both reviewers (100%, kappa = 1.0).

### 2.2 Inclusion and exclusion criteria

Studies were included if they 1) involved empirical research with adolescents and/or adults, 2) used either qualitative or mixed-method approaches, 3) were published in English in peer-reviewed journals and 4) included stand-alone data on patient views or experiences (i.e., patient data could be separated from clinicians or family members) of a DBT intervention, which included a group component (either in person or online). Studies featuring group-only or group-based interventions with additional support (e.g., crisis or one-on-one support) were included because these are increasingly offered in services to enhance the real-world applicability of the findings.

Studies were excluded if they focussed on 1) a specific aspect of DBT only (e.g., experiences of “opposite action” skill only), 2) app use only, or 3) radically open DBT [which differs theoretically from traditional DBT; Gilbert et al. (31)]. As group skills training is a core component of the DBT model, studies lacking this element were excluded to maintain fidelity to the model. Conferences, theses and grey literature were excluded to reduce studies lacking peer review and which were therefore potentially conducted with less standardised scientific rigour.

### 2.3 Methodological quality and risk of bias assessment

The Critical Appraisal Skills Programme (32) for qualitative studies was used to evaluate each study’s methodological quality and risk of bias, by answering its ten questions with “yes”, “no” or “can’t tell”. Questions covered domains such as appropriacy of methodology and ethical considerations. Each item was answered for each study.

To assist with the clearer interpretation of quality and to allow for comparison across studies, the authors decided to assign numerical values to the three possible item responses (No = 0, Partially Agree = 0.5, Yes = 1). Overall methodological quality was then categorised as high (> 8–10), moderate (6–8), or low (≤ 5), allowing for easier reporting of quality [e.g., see Butler et al. and Harries et al. (33, 34)]. Additionally, an independent reviewer quality-rated all included papers, with substantial agreement initially (kappa = 0.65, 93.79%), and almost perfect agreement following discussion of discrepancies (kappa = 0.97, 99.31%).

### 2.4 Data analysis

Data from the “Results” or “Findings” sections, including participant quotes, author interpretations and themes, were extracted into Microsoft Word, transferred to NVivo, and analysed using Thomas and Harden’s (35) thematic synthesis approach. This approach integrates findings from multiple qualitative studies, including mixed-method studies, by identifying and synthesising common themes, providing insights into the acceptability and appropriateness of services or interventions, which can inform policy and practice (36).

Thematic synthesis was conducted in three stages by the first author. First, all extracted text underwent line-by-line coding. Next, similar descriptive codes were grouped inductively, identifying patterns and variations. Finally, analytical themes were developed by reviewing descriptive themes in relation to the study’s aims.

The analysis was undertaken by the first author (AH), with other members of the team (AW and LG) reviewing the credibility and soundness of the themes, in relation to the included studies. This approach was adopted to ensure a thorough approach and minimise bias. All authors approved the final analysis.

### 2.5 Reflexivity statement

The authors were three women and one man, from White European backgrounds. The first author was a trainee clinical psychologist with several years of experience in various clinical roles. The second author was an academic researcher specialising in family and parental mental health, the third was a National Health Service (NHS) clinical psychologist with an interest in DBT, while the final author was a clinical psychologist working both in the NHS and academia. Acknowledging the limitations of an all-white research team, we aimed to minimise potential biases towards a white, Western, or Eurocentric perspective through research team discussions and a rigorous, transparent analytical approach.

## 3 Results

### 3.1 Study characteristics

**Figure 1:**
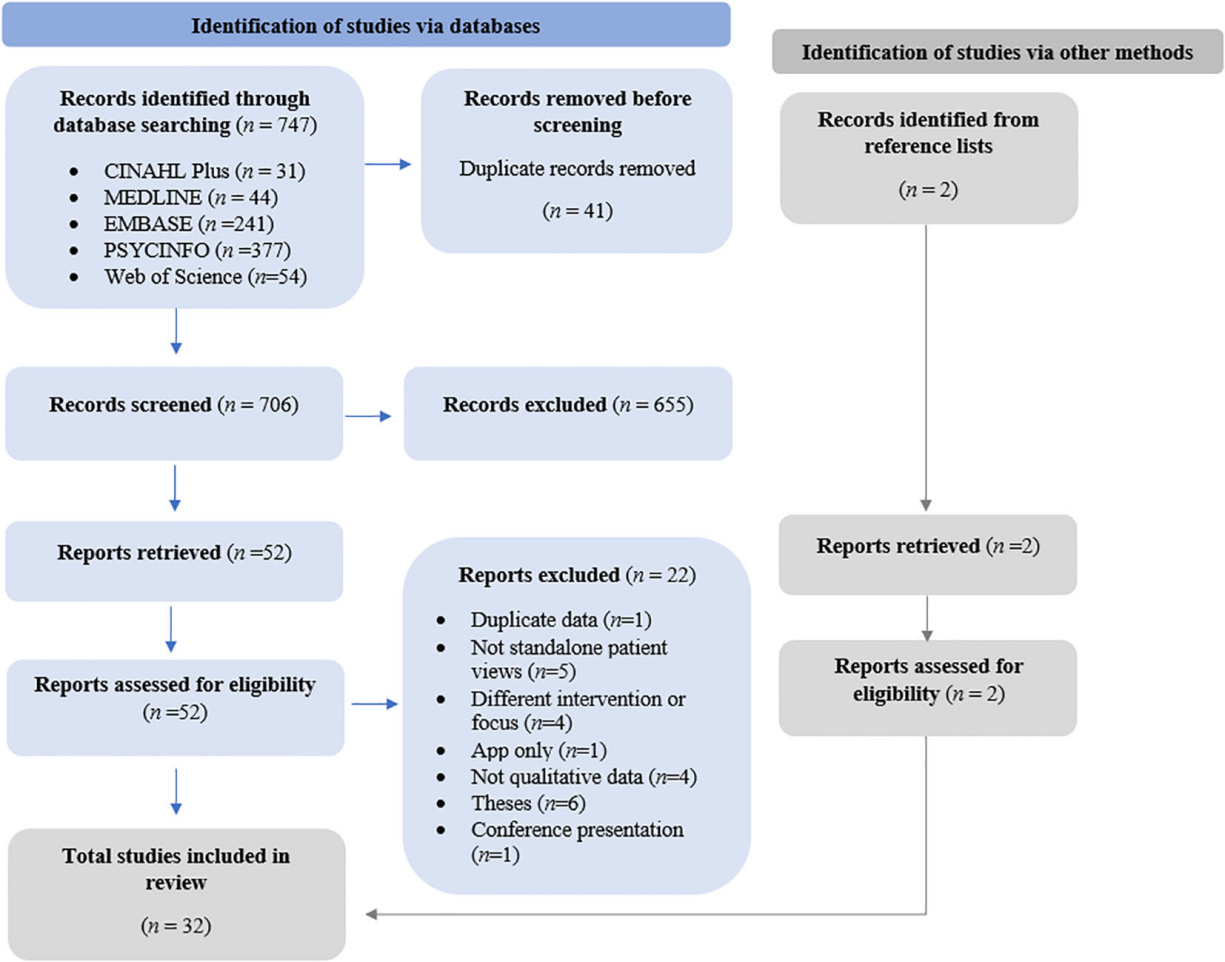
PRISMA diagram.

A total of 747 records were identified by the electronic database search and two records were identified via reference list searching, with 41 identified as duplicates (see **Figure 1** for details). After screening and review, 32 studies were identified as eligible for inclusion and were synthesised, capturing the perspectives of 414 participants on DBT across ten countries over 21 years (2003–2024).

Sample sizes ranged from three to 73 (see **Table 2** for more details). Among studies reporting mental health conditions or clinical populations (n=26), most explored experiences of people with personality disorders or related traits (n=17), including ten specifically on BPD (see **Table 2**). Other participant groups included intellectual disabilities (n=4), perinatal women (n=3), sexual/gender diversity (n=2), anorexia nervosa (n=1), and ADHD (n=1). Five studies took a transdiagnostic approach or did not limit sampling to specific mental health conditions.

**Table 2:** Characteristics of included studies presented in reverse chronological order (adult then adolescent papers).

	Study: authors, year, location	Study aim(s)	Sample description		Intervention	Data collection and analysis	Main themes
			Mental health need/clinical population	Sociodemographic information			
Studies with adult samples							
1	Giles et al. (37) Australia	1) What are the experiences of mothers 3 years after completing the Mother Infant-DBT program? 2) What are the perceived long-term impacts of the MI-DBT program? 3) What improvements could be made to the MI-DBT program or subsequent mental health support based on participant’ experiences?	Mothers with BPD (formal diagnosis or multiple traits)	Sample size: *n=8* Sex: female (*n* = 8) Further demographic information not reported	MI-DBT (38) 24-week group intervention which had been completed 3 years previously. Focus on parenting stress, secure infant attachment and supporting infant emotional regulation.	Interviews Thematic Analysis (39, 40)	1. Keeping my kids in mind 2. Awareness of self 3. Negotiating relationships 4. We want more!
2	Harding et al. (41) UK	To explore lesbian and gay people's experiences of completing a full DBT programme.	Diagnosis not reported	Sample size: *n=8* Sex: female (*n* = 6), male (*n* = 2) Age (years): 22-47, mean age 31.13 Ethnicity: White British (*n* = 5), Mixed (*n* = 2), Not Stated (*n=*1) Employment: Unemployed (*n* = 2), Not Stated (*n* = 1), Part Time & Education (*n* = 3), Employed (*n* = 2) Sexual orientation: gay (*n* = 2), lesbian (*n* = 6)	A full course of standard DBT within the last 2 years. No further details reported.	Interviews Interpretative Phenomenological Analysis (42)	1. The DBT Journey 2. Connections and sense of community during DBT 3. Sexuality both visible and invisible in DBT 4. A gender, sexuality and relationship diverse (GSRD) affirmative future for DBT
3	McColl et al. (43) New Zealand	To understand the experience of the Multidiagnostic Eating Disorder-DBT (MED-DBT) programme for clients with severe and enduring eating disorder symptoms in Aotearoa New Zealand.	Anorexia Nervosa	Sample size: *n* = 7 Sex: female (*n* = 7) Age (years): mean age 31.5 Further demographic information not reported	MED-DBT (44, 45)	Interviews Reflexive Thematic Analysis (39, 46)	1. A valued and valuable experience 2. What sets DBT apart: the value of structure for ED 3. Benefits of comprehensive ED treatment 4. Adaptations for ED 5. Impact of facilitators 6. Continuing and ending treatment 7. Measuring success
4	Francis et al. (47) Australia	To explore the subjective experiences of women who had completed Mother Infant‐DBT (MI-DBT).	Mothers with BPD (formal diagnosis or multiple traits, n not reported)	Sample size: *n* = 13 Sex: female (*n* = 13) Age (years): mean age 31.8 Ethnicity: majority European Australian backgrounds, *n* not reported SES status: moderate-high level of disadvantage	MI-DBT adapted from Linehan (48).2.5hr sessions x 24 weeks. Focus on parenting stress, secure infant attachment and supporting infant emotional regulation.	Interviews Inductive Thematic Analysis (49)	1. Boiling points 2. Emotional literacy 3. Inter-generational transmission 4. Low self-esteem 5. Dealing with disconnect
5	Vasiljevic et al. (50) Sweden	To explore if a brief internet-delivered DBT skills training program with minimal therapist support is acceptable, that it can be administered, useful, and does not do harm for patients with BPD.	BPD/EUPD (*n* = 9) Co-morbid diagnoses: Depression *(n* = 6), anxiety (*n* = 4), ASC (*n* = 1), ADD (*n* = 1), bipolar (*n* = 1), dissociate disorder (*n* = 2), schizophrenia (*n* = 1), substance abuse (*n* = 1)	Sample size: *n* = 9 Sex: female (*n* = 9) Ages (years): 19-37, mean 17.89 Ethnicity not reported Employment: unemployed (*n* = 1), benefits (*n* = 2), part-time employment (*n* = 4), student (*n* = 2)	Abbreviated version of DBT (5, 51), 9 weeks. Each module was administered over 2 weeks. Online group and homework practice.	Semi-structured interviews Content Analysis (52)	1. Working with internet delivered skills training 2. Allocated time 3. The personal contact 4.Personal struggles 5. Outcomes of the skills training program
6	Ohlis et al. (53) Sweden	To explore how former DBT adolescents’ patients experience their treatment, and specifically if there were aspects of the treatment that they retrospectively identify as particularly meaningful, helpful, or unhelpful.	Young adults with self-harming, suicide attempts and features of BPD	Sample size: *n* = 19 Sex: female (*n* = 18), male (*n* = 1) Age (years): 19.2-27.2, mean age 23.1 Ethnicity not reported Employment: employed (*n* = 9), student (*n* = 4), welfare (*n* = 6)	DBT-A (54), (≥40 visits, range 63-254).	Interviews Reflexive Thematic Analysis (39, 55, 56)	1. The need to be seen, listened to and believed in. 2. The importance of teamwork between patient and therapist. 3. The value of group and structure. 4. Therapy as lifesaving and life changing. 5. The risks of feeling misplaced. 6. The risks of abrupt endings
7	Gillespie et al. (57) Ireland	How do individuals who report having benefitted from DBT report their lives, their coping, and their approaches to problems at long-term follow-up? What, if any, are the DBT-related processes which these participants identify at long-term follow-up as having affected their lives? Do individuals who feel they benefitted from DBT at the time continue to report benefits at two or more years post-completion?	BPD	Sample size: *n* = 12 Sex: female (*n* = 9), male (*n* = 3) Ages (years): 25-73, mean age 46.08 Ethnicity not reported Employment: employed (*n* = 6)	DBT (Linehan, 1993a; 1993b). Either 6- or 12-month programme. Included 1–1 and group sessions weekly, plus phone coaching and consultation meetings.	Interviews Thematic Analysis (39, 56, 58)	1. DBT is life changing but not a magic wand: a foundation to build from 2. “And to have that control is, oh god, it’s like a miracle”: responding versus reacting to problems 3. “I have really good friends now, which is something that I probably would have never really of had”: meaningful and healthier relationships with others
8	Rouski et al. (59) UK	Aimed to explore young people’s perspectives of Skills for Living Service (including DBT skills group)	Care leavers, diagnosis not reported	Sample size: *n* = 10 Sex: male (*n* = 3), female (*n* = 7) Ages (years): 17-22 Ethnicity not reported Further demographic information not reported	DBT Skills Group, 10–12-week programme (60). Weekly sessions last 2.5 hours including a break and cover all four core modules.	Semi-structured interviews Thematic Analysis (39)	1. Initial apprehension and reluctance to participate 2. Connection, understanding and validation 3. Confidence with social skills 4. Emotional acceptance and self-soothing
9	Barnicot et al. (61) UK	To establish evidence on common and unique, helpful, and unhelpful, treatment processes for DBT and MBT.	Personality disorder (BPD *n* = 71, Other PD *n* = 2)	Sample size: *n* = 73 Sex: female (*n* = 56), male (*n* = 17) Ages (years): mean age 30.9 Ethnicity: White British (*n* = 42), White Other (*n* = 5), Black (*n* = 7), South Asian (*n* = 9), mixed (*n* = 10) Employment: full-time (*n* = 7), part-time (*n* = 11), sick leave (*n* = 3), unemployed (*n* = 52)	DBT (12-month programme), consisting of weekly individual and group therapy, as well as telephone skills coaching (reference not reported).	Interviews Thematic Analysis (39)	1. Support and Insight from 1–1 sessions. 2. Feeling understood and gaining alternative perspectives from other group members. 3. Becoming more self-aware. 4. Not reacting impulsively. 5. Questioning thoughts and assumptions. 6. Behavioural techniques for reducing distress. 7. Communicating more effectively. 8. Difficulties in the therapeutic relationship. 9. Difficulties interacting with other group members. 10. Painful introspection.
10	Pearson et al. (62) UK	To explore subjective experiences of DBT in a community setting for people with ID.	Intellectual disability (*n* = 11) Co-morbid diagnoses: EUPD (*n* = 6), EUPD symptoms (*n* = 4). Depression (*n* = 1)	Sample size: *n=11* Sex: female (*n* = 8), male (*n* = 3) Ages (years): 26-52, mean age 38 Ethnicity: British (*n* = 11) Further demographic information not reported	DBT (all had weekly group and 1:1 therapy, and team consultation; 9 had telephone coaching). Reference not reported.	Interviews Interpretative Phenomenological Analysis (63)	1. Experience of power 2. Differences in therapy contexts 3. Experience of a positive therapeutic relationship 4. A new way of being
11	Greaves et al. (64) UK	Hypothesised that community perinatal DBT skills groups that included babies would reduce distress and improve emotional regulation.	Perinatal women with various diagnoses, *n* not reported	Sample size: *n* = 7 Sex: female (*n* = 7) Further demographic information not reported	DBT (5), weekly 2hr groups lasting either 12 or 14 weeks, with telephone coaching and team consultation. Perinatal adaptations made, reference not reported.	Semi-structured interviews Thematic Analysis (39)	1. Self as Mother 2. Shared Experience 3. The impact of babies
12	Austin et al. (65) Denmark	To describe the experiences of participants while using a mobile phone app that was designed to enhance and support dialectical behaviour therapy for personality disorders.	Personality disorder (BPD *n* = 6, other *n* = 2) Co-morbid diagnoses: anxiety, ADHD, other personality disorders, *n* not reported	Sample size: *n* = 8 Sex: female (n=8) Age (years): 25-35 Ethnicity not provided Further demographic information not reported	DBT (20x 2hr group sessions, plus 1–1 therapy fortnightly, supported by mobile app use). Reference not reported.	Interviews Thematic Analysis (39)	1. An overall positive experience of using the app—participants 2. The app provided a common source of information for patient and therapist interactions
13	Lakeman and Emeleus (66) Australia	Consider what elements contribute to its effectiveness, or the characteristics of those who complete the programme and achieve recovery.	Young adults with BPD	Sample size: *n* = 6 Sex and ethnicity not reported Age (years): 17.4-25.3, mean age 20.4 Further demographic information not reported	DBT (5) including weekly individual therapy, 20 x 3hr group sessions, telephone coaching and team consultation.	Semi structured interviews Constant comparative analysis (67)	1. ‘Becoming a cheerleader for DBT’ 2. ‘Learning the language of DBT and consolidation of skills’
14	Harned and Schmidt (68) USA	The primary aim of the present study was to explore DBT consumers’ perspectives on DBT + DBT Prolonged Exposure	BPD, *n* not reported Co-morbid diagnosis: PTSD (*n* = 15)	Sample size: *n* = 19 Sex: female (*n* = 19) Age (years): mean age 35.3 Ethnictiy: White (*n* = 9), African American (*n* = 7), multiracial (*n* = 2), Hispanic (*n* = 2) Further demographic information not reported	DBT (weekly individual and group skills training, team consultation and telephone coaching). Both agencies considering DBT + DBT Prolonged Exposure. Reference not reported.	Focus groups Qualitative Content Analysis (69)	1. Barriers 2. Facilitators
15	Hewitt et al. (70) UK	To report quantitative and qualitative follow-up data from three participants who attended a Dialectical Behaviour Therapy (DBT) group for people with an intellectual disability.	Intellectual disability (*n* = 3) Co-morbid diagnoses: EUPD (*n* = 1) ASC (*n* = 1) OCD (*n* = 1)	Sample size: *n* = 3 Sex: female (*n* = 3), male (*n* = 1) Age (years): 30-50 Ethnicity: White British (*n* = 2), White Other (*n* = 1) Further demographic information not reported	Adapted 18 session DBT programme (1, 71), see Crossland et al. (72)for full description.	Interviews Thematic Analysis (39)	1. Remembering vs forgetting 2. Thinking about the future 3. Group as a positive or negative experience 4. Personalisation and adaption 5. Continuity and impact of the group
16	Childs-Fegredo and Fellin (73) UK	To explore the client experience of a 12-week transdiagnostic DBT group programme in a private psychiatric hospital.	BPD (*n* = 3) Bipolar (*n* = 1) Depression (*n* = 1)	Sample size: *n* = 5 Sex: female (*n* = 4), male (*n* = 1) Further demographic information not reported	Adapted DBT programme (12 weeks – weekly group and 1:1 sessions, telephone coaching and team consultation). Reference not reported.	Semi structured interview Interpretative Phenomenological Analysis (63, 74)	1. Pre DBT: ‘Crisis & Desperation’ 2. In-session: ‘Belonging’ 3. The Real World: ‘Living’
17	Russell and Siesmaa (75) UK	To qualitatively explore the experiences of high risk and adult male forensic clients (diagnosed with BPD) and antisocial personality disorder (ASPD)) in a forensic adapted version of DBT.	BPD and anti-social personality disorder (*n* = 6).	Sample size: *n* = 6 Sex: male (*n* = 6) Age (years): 34-61, mean age 47 Ethnicity not provided. Further demographic information not reported	Adapted forensic DBT programme (2hr groups weekly, 1–1 session weekly, telephone consultation and weekly therapist consultations). Adaptations included addition of violent behaviour and language/examples being adapted to male forensic population. Reference not reported.	Semi-structured interview Thematic Analysis (39)	1. Processes 2. Treatment Outcomes
18	Thomson and Johnson (76) UK	To look at women’s experience of undertaking this newly introduced therapy by asking them to share and elaborate on their experience.	Intellectual disability and BPD (*n* = 7)	Sample size: *n* = 7 Sex: female (*n* = 7) Further demographic information not reported	Reference not reported.	Semi-structured interview Interpretative Phenomenological Analysis (77)	1. How you do DBT 2. What we think about DBT 3. Using DBT
19	Crossland, Hewitt and Walden (72) UK	To evaluate a community run DBT group through collecting quantitative and qualitative data.	Intellectual Disability (*n* = 4) Co-morbid diagnoses: EUPD (*n* = 2), ASC (*n* = 1), OCD (*n* = 1), Depression (*n* = 1)	Sample size: *n* = 4 Sex: female (*n* = 3), male (*n* = 1) Age (years)= 24-48 Ethnicity: White other (*n* = 1), White British (*n* = 3) Further demographic information not reported	18-week modified DBT skills training group based on Linehan (2), with adapted materials from Ingamells and Morrissey (71).	Semi-structured interview Thematic Analysis (39)	1. Interpersonal Relationships 2. Group Boundaries 3. Making Theory-Practice Links 4. Remembering our new skills 5. Generalising skills outside the group 6. Suggestions for improvements
20	Roscoe, Petalas, Hastings and Thomas (78) UK	To explore the views and experiences of female inpatients, with a diagnosis of a personality disorder and an ID, about dialectical behaviour therapy (DBT)	Intellectual disability (*n* = 10) Co-morbid diagnoses: BPD (*n* = 9) ADHD (*n* = 1) Dependent PD (*n* = 1)	Sample size: *n* = 10 Sex: female (*n* = 10) Age (years): 19-57, mean age 44.9 Ethnicity: White British (*n* = 10) Further demographic information not reported	Based on Linehan (2) DBT. Adaptations made for LD and PD – reference not reported. Intervention ranged from 3–23 months.	Semi structured interview Interpretative Phenomenological Analysis (63)	1. Understanding DBT 2. DBT as helpful and beneficial 3. Engagement with DBT process
21	McCay et al. (79) Canada	To evaluate a 12-week DBT intervention to determine whether or not the intervention would be effective in reducing emotional distress and maladaptive coping mechanisms (e.g. addiction), as well as promoting positive relationships, resilience and overall functioning among street-involved youth.	Street-involved youth. Diagnosis not reported.	Sample size: *n* = 30 Sex: male (*n* = 13), female (*n* = 17) Age (years): mean age 21.4 Ethnicity not reported Further demographic information not reported	DBT-A (54). 12 weeks of individual and group sessions, with telephone coaching. Shortened to 12 weeks given engagement challenges of population. No parent group given not appropriate for many in this population.	Semi-structured interviews Thematic Analysis (80)	1. Impetus for DBT 2. Experience of DBT 3. Impact of DBT
22	McSherry et al. (81) Ireland	The study examined service users' perspective on the effectiveness of an adapted DBT programme, delivered within a community adult mental health setting.	BPD	Sample size: *n* = 8 Sex: female (*n* = 6), male (*n* = 2) Age (years): 32–55 Ethnicity not reported Further demographic information not reported	Adapted DBT in CMHT (27 weeks x 2hr groups, 1–1 session, telephone consultation and weekly therapist consultations). Reference not reported.	Semi-structured interviews and focus groups Thematic Analysis (39)	1. Evaluation of therapy 2. Treatment Impact
23	McFetridge and Coakes (82) UK	To further examine the longer-term effectiveness of therapeutic approaches for clients with BPD, particularly those who are unlikely to remit	BPD	Sample size: *n* = 11 Demographic information not reported	DBT (8–12-month programme) – reference not reported.	Semi-structured questionnaire and video accounts of experiences Interpretive Phenomenological Analysis (83)	1. Changes in sense of identity 2. Changes in life 3. Changes in thinking
24	Hodgetts, Wright and Gough (84) UK	To explore clients’ experiences of DBT and the impact this treatment has on their lives.	BPD	Sample size: *n* = 5 Sex: male (*n* = 2), female (*n* = 3) Age (years): 24-48 Ethnicity not reported Further demographic information not reported	DBT intervention for 6–12 months. Reference not reported.	Semi-structured interviews Interpretative Phenomenological Analysis (42)	1. Joining a DBT programme 2. Experiences of DBT 3. Evaluation of DBT
25	Cunningham, Wolbert and Lillie (85) USA	Goal of understanding, from the perspective of the client, what is effective about DBT and why.	BPD	Sample size: *n* = 14 Sex: female (*n* = 14) Age (years): 23-61, mean age 38.7 Ethnicity not reported Further demographic information not reported	DBT (2). Programme time ranged from 6–36 months, involving weekly individual and group training as well as telephone coaching and team consultation.	Open-ended semi-structured interviews Interpretative Analysis (reference not reported).	1. Individual Therapy 2. Skills Training 3. Skills Coaching 4. Relationships 5. Level of suffering 6. Level of hope
26	Perseius, Ojehagen, Ekdahl, Asberg and Samuelsson (86) Sweden	To describe patients ‘and therapists’ perception of receiving and giving DBT treatment.	BPD Co-morbid diagnoses: Depression (*n* = 9), anxiety (*n* = 9), eating disorders (*n* = 3), social phobia (*n* = 2), substance misuse (*n* not reported).	Sample size: *n* = 10 Sex: female (*n* = 10) Age (years): 22-49, median age 27 Ethnicity not reported Further demographic information not reported	DBT (reference not reported), with participants engaged for 12 months or longer.	Interviews Qualitative Content Analysis (87)	1. The therapy effect 2. The effective components of therapy 3.Perceptions of psychiatric care before entering DBT
Studies with adolescent samples							
27	Camp et al. (88) UK	1. Whether GSM adolescents receiving DBT experienced difficulties relating to their sexual and gender identity in their everyday lives, and which of these they thought were important to include in DBT (whether it was targeted or not). 2. Adolescents’ perceptions of how DBT met their needs as GSM individuals and how effective the approach was for supporting them with GSM-associated difficulties.	Gender-and sexuality-minoritised adolescents. All participants under Child and Adolescent Mental Health Services	Sample size: *n* = 14 Gender: female (*n* = 6), non-binary (*n* = 4), gender fluid (*n* = 1), questioning (*n* = 3) Ages (years): 14-18 Ethnicity: White British (*n* = 11), White Irish (*n* = 1), Black British (*n* = 1), mixed White & Asian *n* = 1) Sexual orientation: bisexual (*n* = 7), gay/lesbian (*n* = 3), pansexual (*n* = 2), queer (*n* = 1), aromantic (*n* = 1)	DBT model for adolescents (89, 90), minimum of 6 months in treatment. Includes 1–1 and group sessions weekly, telephone coaching and parent group. Parent intervention informed by Fruzzetti (91) and delivered separately in this service context (88).	Interviews Reflexive thematic analysis (40)	1. Identity 2. Impact of others 3. Space for sexual and gender identity in DBT
28	Bock et al. (92) Germany	Assessing an online DBT-A skills group, considering up and downsides, and form a basis for advancement of this treatment provision.	BPD, Depression, Anxiety, *n* not reported	Sample size: *n* = 5 Sex: mainly female, *n* not reported. Ages (years): 14-18 Ethnicity not reported Further demographic information not reported.	Group DBT-A (93) delivered by video call. Length not reported.	Focus groups Qualitative Content Analysis (94)	1. Perceived impact of pandemic 2. Technical issues 3. Perceived benefits
29	Ramzan, Dixey and Morris (95) UK	What challenges and benefits have adolescents encountered in virtual DBT-A? How acceptable have they found doing virtual DBT-A? What suggestions do they have for improving the experience of virtual DBT-A?	Self-harming behaviours, emotion dysregulation and features of BPD.	Sample size: *n* = 13 Sex not reported. Ages (years): 14-18 Ethnicity: White (*n* = 7), mixed (*n* = 1), other/not stated (*n* = 5) Sexual orientation: heterosexual (*n* = 3), bisexual (*n* = 4), other (*n* = 1), not stated (*n* = 2), missing (*n* = 3)	DBT-A (54), 8–12-month programme. Individual and group weekly sessions, with telephone coaching for adolescents and parents, and a weekly parent group for the first 6 months. Weekly team consultation meetings.	Qualitative survey Thematic Analysis (40)	1. Sense of loss 2. Feeling uncontained 3. Benefits of virtual DBT
30	Ratnaweera, Hunt and Camp (96) UK	1. To evaluate the acceptability of the treatment programme. 2. To explore service users’ retrospective expectations of DBT 3. To investigate perceived benefits of the treatment programme, 4. To explore suggestions for improvements and modifications of the treatment programme 5. To explore service user’s experience of using DBT skills to manage their difficulties and investigate the type of skills used outside of therapy.	BPD symptoms (including self-harm 2+ episodes of self-harm in past 6 months, and symptoms in 5 domains of BPD)	Sample size: *n* = 18 Gender: female (*n* = 16), non-binary (*n* = 2) Age (years): 14.3-17.6, mean age 16.52 Ethnicity: White (*n* = 11), Black (*n* = 1), mixed (*n* = 4), other White (*n* = 1), other (*n* = 1) Sexuality: heterosexual (*n* = 8), bisexual (*n* = 2), gay/lesbian (*n* = 2), demisexual (*n* = 1), queer (*n* = 1), pansexual (*n* = 1), asexual (*n* = 1), not stated (*n* = 2), missing (*n* = 1)	DBT based on Linehan (1), Miller and Rathus (93) and Miller, Rathus and Linehan (89). 8–12-month programme comprised of weekly individual and telephone coaching, plus 6 months of group sessions weekly, and a parent’s group.	Interviews Thematic Analysis (39).	1. A new way of living 2. Better understanding of self 3. New skills 4. Person-centred approach 5. Relationships with others
31	Meyer, Ramklint, Oster and Isaksson (97) Sweden	To explore how adolescents with ADHD experience participating in a structured skills training group program based on dialectical behavioural therapy	ADHD (n=13), ADD (n=5), ADHD (NOS) (n=2)	Sample size: *n* = 20 Sex: female (*n* = 12), male (*n* = 8) Age (years): 15-18, mean age 16.3 Ethnicity not reported Further demographic information not reported	DBT group programme (98, 99). 2hr x 14-week sessions. Some adaptations for younger age range, reference not reported.	Semi-structured interviews Qualitative Content Analysis (100)	1. A need to belong 2. Need to be an active participant in one’s own treatment
32	Pardo et al. (101) Spain	To examine the subjective experience of adolescents with behavioural issues who have completed DBT skills training group,	Personality disorder (*n* = 15), BPD features (*n* = 2), adjustment disorder (*n* = 14), impulse control disorder (*n* = 3), conduct disorder (*n* = 1), disruptive behaviour disorder (*n* = 1), intermittent explosive disorder (*n* = 1)	Sample size: *n* = 20 Sex: female (*n* = 16), male (*n* = 4) Ages (years): 14-17, mean age 15.40 Ethnicity not reported Further demographic information not reported	DBT skills training group (16 x weekly sessions). Reference not reported.	Focus Groups Grounded Theory (102)	1. Experience of illness 2. Motivation for therapy 3. Experience of therapy 4. Results of the therapy

Eleven studies focussed on female perspectives, one on male perspectives, 16 reported mixed-sex views, and four studies did not provide this information. Most studies examined adult experiences (n=26), while six focussed on adolescents or young people (aged 14-27). Ethnicity was rarely reported, with only 12 studies providing data.

Of the 32 studies, 22 used a qualitative approach, while ten employed mixed-methods. Interviews were the most common data collection method (n=26), followed by focus groups (n=2). Three studies combined interviews with focus groups or questionnaires, and one used a qualitative survey. Thematic Analysis was the most frequently used analysis method (n=17), followed by Interpretative Phenomenological Analysis (IPA) (n=7), Content Analysis (n=5), other approaches (n=2) and Grounded Theory (n=1).

### 3.2 Methodological quality of the 32 included studies

Thirty-one of the 32 studies were assessed as having high methodological quality, while only one was rated to have moderate methodological quality (82). This study involved a qualitative survey. The methodological quality of included studies is detailed in **Table 3**.

**Table 3:** Methodological quality of the 32 included studies.

	Study: authors and year	Was there a clear statement of the aims of the research?	Is a qualitative methodology appropriate?	Was the research design appropriate to address the aims of the research?	Was the recruitment strategy appropriate to the aims of the research?	Was the data collected in a way that addressed the research issue?	Has the relationship between researcher and participants been adequately considered?	Have ethical issues been taken into consideration?	Was the data analysis sufficiently rigorous?	Is there a clear statement of findings?	How valuable is the research?	Quality appraisal (total score)
1	Giles et al. (37)	Yes (1)	Yes (1)	Yes (1)	Yes (1)	Yes (1)	Yes (1)	Yes (1)	Yes (1)	Yes (1)	Yes (1)	High (10)
2	Harding et al. (41)	Yes (1)	Yes (1)	Yes (1)	Yes (1)	Yes (1)	Yes (1)	Yes (1)	Yes (1)	Yes (1)	Yes (1)	High (10)
3	McColl et al. (43)	Yes (1)	Yes (1)	Yes (1)	Yes (1)	Yes (1)	PA (0.5)	Yes (1)	Yes (1)	Yes (1)	Yes (1)	High (9.5)
4	Francis et al. (47)	Yes (1)	Yes (1)	Yes (1)	Yes (1)	Yes (1)	PA (0.5)	Yes (1)	PA (0.5)	Yes (1)	Yes (1)	High (9)
5	Ohlis et al. (51)	Yes (1)	Yes (1)	Yes (1)	Yes (1)	Yes (1)	Yes (1)	Yes (1)	Yes (1)	Yes (1)	Yes (1)	High (10)
6	Camp et al. (88)	Yes (1)	Yes (1)	Yes (1)	Yes (1)	Yes (1)	Yes (1)	Yes (1)	Yes (1)	Yes (1)	Yes (1)	High (10)
7	Vasiljevic et al. (50)	Yes (1)	Yes (1)	PA (0.5)	Yes (1)	Yes (1)	PA (0.5)	Yes (1)	Yes (1)	Yes (1)	Yes (1)	High (9)
8	Bock et al. (92)	Yes (1)	Yes (1)	Yes (1)	Yes (1)	Yes (1)	PA (0.5)	Yes (1)	PA (0.5)	Yes (1)	Yes (1)	High (9)
9	Gillespie et al. (57)	Yes (1)	Yes (1)	Yes (1)	Yes (1)	Yes (1)	Yes (1)	Yes (1)	Yes (1)	Yes (1)	Yes (1)	High (10)
10	Ramzan et al. (95)	Yes (1)	Yes (1)	Yes (1)	Yes (1)	Yes (1)	Yes (1)	Yes (1)	Yes (1)	Yes (1)	Yes (1)	High (10)
11	Rouski et al. (59)	Yes (1)	Yes (1)	Yes (1)	Yes (1)	Yes (1)	PA (0.5)	PA (0.5)	Yes (1)	Yes (1)	Yes (1)	High (9)
12	Barnicot et al. (61)	Yes (1)	Yes (1)	Yes (1)	Yes (1)	Yes (1)	Yes (1)	Yes (1)	Yes (1)	Yes (1)	Yes (1)	High (10)
13	Ratnaweera et al. (96)	Yes (1)	Yes (1)	Yes (1)	Yes (1)	Yes (1)	No (0)	Yes (1)	Yes (1)	Yes (1)	Yes (1)	High (9)
14	Pearson et al. (62)	Yes (1)	Yes (1)	Yes (1)	Yes (1)	Yes (1)	Yes (1)	Yes (1)	Yes (1)	Yes (1)	Yes (1)	High (10)
15	Greaves et al. (64)	Yes (1)	Yes (1)	Yes (1)	Yes (1)	Yes (1)	Yes (1)	Yes (1)	Yes (1)	Yes (1)	Yes (1)	High (10)
16	Meyer et al. (97)	Yes (1)	Yes (1)	Yes (1)	Yes (1)	Yes (1)	PA (0.5)	Yes (1)	Yes (1)	Yes (1)	Yes (1)	High (9.5)
17	Austin et al. (65)	Yes (1)	Yes (1)	Yes (1)	Yes (1)	Yes (1)	No (0)	Yes (1)	Yes (1)	Yes (1)	Yes (1)	High (9)
18	Pardo et al. (101)	Yes (1)	Yes (1)	Yes (1)	Yes (1)	Yes (1)	No (0)	Yes (1)	Yes (1)	Yes (1)	Yes (1)	High (9)
19	Lakeman and Emeleus (66)	Yes (1)	Yes (1)	Yes (1)	Yes (1)	Yes (1)	PA (0.5)	Yes (1)	Yes (1)	PA (0.5)	Yes (1)	High (9)
20	Harned and Schmidt (68)	Yes (1)	Yes (1)	Yes (1)	Yes (1)	Yes (1)	PA (0.5)	Yes (1)	Yes (1)	Yes (1)	Yes (1)	High (9.5)
21	Hewitt et al. (70)	Yes (1)	Yes (1)	Yes (1)	Yes (1)	Yes (1)	Yes (1)	Yes (1)	Yes (1)	Yes (1)	Yes (1)	High (10)
22	Childs-Fegredo and Fellin (73)	Yes (1)	Yes (1)	Yes (1)	Yes (1)	Yes (1)	Yes (1)	Yes (1)	Yes (1)	Yes (1)	Yes (1)	High (10)
23	Russell and Siesmaa (75)	Yes (1)	Yes (1)	Yes (1)	Yes (1)	Yes (1)	Yes (1)	Yes (1)	Yes (1)	Yes (1)	Yes (1)	High (10)
24	Thomson and Johnson (76)	Yes (1)	Yes (1)	Yes (1)	Yes (1)	Yes (1)	PA (0.5)	Yes (1)	Yes (1)	Yes (1)	PA (0.5)	High (9)
25	Crossland et al. (72)	Yes (1)	Yes (1)	Yes (1)	Yes (1)	Yes (1)	No (0)	Yes (1)	Yes (1)	Yes (1)	Yes (1)	High (9)
26	Roscoe et al. (78)	Yes (1)	Yes (1)	Yes (1)	Yes (1)	Yes (1)	Yes (1)	Yes (1)	Yes (1)	Yes (1)	Yes (1)	High (10)
27	McCay et al. (79)	Yes (1)	Yes (1)	Yes (1)	Yes (1)	Yes (1)	No (0)	Yes (1)	Yes (1)	Yes (1)	Yes (1)	High (9)
28	McSherry et al. (81)	Yes (1)	Yes (1)	Yes (1)	Yes (1)	Yes (1)	PA (0.5)	PA (0.5)	Yes (1)	Yes (1)	Yes (1)	High (9)
29	McFetridge and Coakes (82)	PA (0.5)	Yes (1)	Yes (1)	Yes (1)	Yes (1)	No (0)	No (0)	Yes (1)	PA (0.5)	PA (0.5)	Moderate (6.5)
30	Hodgetts et al. (84)	Yes (1)	Yes (1)	Yes (1)	Yes (1)	Yes (1)	No (0)	Yes (1)	Yes (1)	Yes (1)	Yes (1)	High (9)
31	Cunningham et al. (85)	Yes (1)	Yes (1)	Yes (1)	Yes (1)	Yes (1)	PA (0.5)	PA (0.5)	Yes (1)	Yes (1)	PA (0.5)	High (8.5)
32	Perseius et al. (86)	Yes (1)	Yes (1)	Yes (1)	Yes (1)	Yes (1)	PA (0.5)	Yes (1)	Yes (1)	Yes (1)	Yes (1)	High (9.5)
	Total	98.44%	100%	98.44%	100%	100%	59.34%	92.19%	96.90%	96.90%	95.31%	

PA Partially agree (indicated by orange shading). Green indicates ‘Yes’ and Red indicates ‘No’. High >8-10, moderate (6-8), low (≤5).

Thirteen studies received the maximum quality rating: a rating of “yes” for all items (or a score of 10/10). Notably, only 13 of the 32 included studies considered the researcher-participant relationship sufficiently, meaning that item six of the CASP was rated the lowest across studies. Four studies had not sufficiently stated if ethical issues were taken into consideration (59, 81, 82, 85).

### 3.3 Thematic synthesis

**Figure 2:**
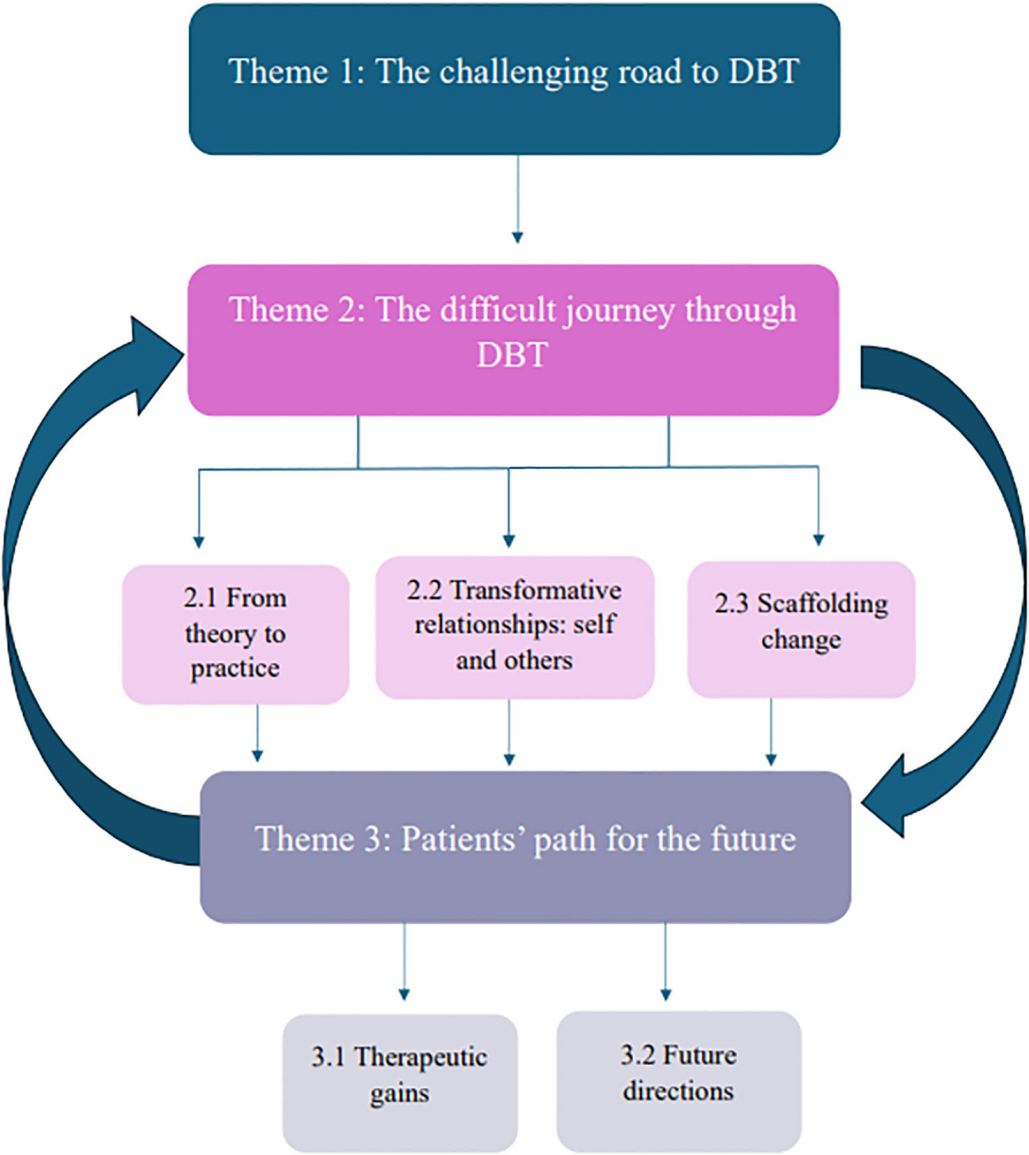
Conceptual diagram depicting themes and sub-themes.

Three over-arching themes, with six sub-themes were developed representing how people experienced DBT: 1) *the challenging road to DBT*, 2) *the difficult journey through DBT* and 3) *patients’ path for the future*. **Figure 2** illustrates the themes relationship to each other. Illustrative quotes are provided below each theme, with additional data presented in **Table 4**. **Table 5** presents a matrix of themes, illustrating which themes were present in each study.

**Table 4:** Thematic structure with additional illustrative quotes.

Theme	Additional illustrative quotes
Theme 1: The challenging road to DBT	
	**“** *Then it just became like this cycle that I was living in that really didn’t bear any fruits”* (McCay et al. (79), p.194). *“I find that with CBT sometimes what you end up doing is putting a plaster over it and just leaving it and then it reopens again.”* (Harding et al. (41), p. 8).
Theme 2: The difficult journey through DBT	
Sub-theme 2.1: From theory to practice	“*I’m not as impulsive as I used to be”*, (Ratnaweera et al. (96), p.6). *“I always go and use my box all the time to calm myself down”* (Rouski et al. (59), p.462). *“I have learnt about myself, and I found out why I act in the way I do”* (Austin et al. (65), p.7). *“And DBT is very practical, it teaches you what to do with your feelings” (*Cunningham et al. (85), p.255).
Sub-theme 2.2: Transformative relationships - self and others	*“I think that sitting around and thinking happy thoughts hasn’t worked for me so far. And the relief about DBT is that [ … ], you don’t have to sit around thinking happy thoughts, it’s ok that you are feeling a bit shit, actually”* (Childs-Fegredo & Fellin (73), p.323). *“It comes down to acceptance because then I don’t fight with myself over my illness and then I’m able to be more aware of my emotional state when I react, so I don’t react straight away”* (Giles et a (37)., p.7).
Sub-theme 2.3: Scaffolding and supporting change	*“Thanks to the telephone coaching you never feel left all by yourself”* (Perseius et al. (86), p.223). “H*e made us go up to the blackboard, to explain it, participate [ … ] he made us study the theory [ … ] it was different from other groups”*, Pardo et al. (101), p.5).
Theme 3: Patients’ path for the future	
Sub-theme 3.1: Therapeutic gains	*“I would never want to take those skills away [ … ]. It just really shows that you can actually calm yourself down, you can actually make relationships, you can actually respect yourself and self-sooth yourself”* (McCay et al. (79), p.196).
Sub-theme 3.2: Future directions	*“I think, would be very nice to get a refresher course on … I suppose it’s like everything, if you learn a new skill and if you’re only using half of it, well you are going to forget the other half.”* (Gillespie et al. (57), p.6).

**Table 5:** Matrix of the 32 included studies and identified themes.

	Theme 1: The challenging road to DBT	Theme 2: The difficult journey through DBT			Theme 3: Patients’ path for the future	
		Content	Processes that supported change	Delivery and format	Therapeutic gains	Future suggestions for therapy
Studies including adult samples						
Giles et al. (37)	✓	✓	✓		✓	✓
Harding et al. (41)	✓	✓	✓	✓	✓	✓
McColl et al. (43)	✓	✓	✓	✓	✓	
Francis et al. (47)	✓	✓	✓	✓	✓	✓
Ohlis et al. (53)	✓	✓	✓	✓	✓	
Vasiljevic Isaksson et al. (50)		✓	✓	✓	✓	✓
Gillespie et al. (57)	✓	✓	✓	✓	✓	✓
Rouski et al. (59)	✓	✓	✓	✓	✓	
Barnicot et al. (61)	✓	✓	✓	✓	✓	
Pearson et al. (62)	✓	✓	✓	✓	✓	
Greaves et al. (64)		✓	✓	✓	✓	
Austin et al. (65)		✓	✓	✓		
Lakeman and Emeleus (66)	✓	✓	✓	✓	✓	
Harned and Schmidt (68)	✓	✓	✓	✓	✓	✓
Hewitt et al. (70)		✓	✓	✓	✓	✓
Childs-Fegredo and Fellin (73)	✓	✓	✓	✓	✓	
Russell and Siesmaa (75)	✓	✓	✓	✓	✓	
Thomson and Johnson (76)	✓	✓	✓	✓	✓	✓
Crossland, Hewitt and Walden (72)		✓	✓	✓		✓
Roscoe, Petalas, Hastings and Thomas (78)	✓	✓	✓	✓	✓	
McCay et al. (79)	✓	✓	✓	✓	✓	
McSherry et al. (81)	✓	✓	✓	✓	✓	✓
McFetridge and Coakes (82)		✓	✓		✓	
Hodgetts, Wright and Gough (84)	✓	✓	✓	✓	✓	
Cunningham, Wolbert and Lillie (85)	✓	✓	✓	✓	✓	✓
Persieus et al. (86)	✓	✓	✓	✓	✓	
Studies including adolescent samples						
Camp et al. (88)	✓	✓	✓	✓	✓	✓
Bock et al. (92)		✓	✓	✓		
Ramzan et al. (95)		✓	✓	✓		
Ratnaweera, Hunt and Camp (96)		✓	✓	✓	✓	✓
Meyer et al. (97)		✓	✓	✓	✓	✓
Pardo et al. (101)	✓	✓	✓	✓	✓	

#### 3.3.1 Theme 1: the challenging road to DBT

This theme focussed on the constellation of participant experiences prior to DBT, which motivated individuals to engage with the intervention. It describes how difficult journeys characterised by physical and relational trauma, adversity, isolation, and the subsequent harmful coping patterns that emerged led people to DBT.

Across study samples of adults and adolescents, participants described various traumas, including physical and sexual abuse, bullying and hospitalisation for mental health needs. A sense of loneliness (*“being the only one in the world feeling this way”*, Ohlis et al. (53), p.418), low self-esteem (*“I always used to doubt myself and tell myself I wasn’t good enough”*, Rouski et al. (59), p.460) and emotional overwhelm (*“I tend to internalise everything and then things just boil over”*, Francis et al. (47)., p.1250) were common psychological sequelae of trauma, which, linked with harmful coping patterns, often appeared to perpetuate distress. According to participant narratives, further impacts of these traumas, including their impact on functioning (“*since then I have no idea how to be an adult really because I just behave like a child when I am stressed”*, Francis et al. (47), p.1252), a desire to be relieved from the pain (“*it was* sp*iralling down, downwards … I was like, why is this happening, like how do I get out of this*?”, McCay et al. (79), p.194) and a desire not to be a victim (“*I want to take it and show those morons who abused me that I am the warrior, that I am the victor”*, Cunningham et al. (85), p.248) encouraged individuals to seek psychological support and access DBT.

Participant narratives also indicated that increased self-awareness, combined with internally (*“I just don’t want to be like this for the rest of my life”*, Hodgetts et al. (84), p.174) and externally “*I really really want to prove to them [ … ] that I can actually do this”*, Russell & Siesmaa (75), p.51) focussed desires to change these patterns facilitated initial engagement, alongside sustaining participation with DBT. Furthermore, self-recognition of functioning abilities and attempts to cope were reported to have a negative impact on their lives, and to influence motivation and commitment to the intervention. Many individuals described that they had previously engaged in therapy without success, likely because first-line interventions did not address their complex needs, seemingly leading to an increased willingness to engage with DBT.

Overall, this theme illustrates the difficult and often traumatic experiences that led individuals to engage with DBT, highlighting how a combination of personal struggles, harmful coping mechanisms, and a deep desire for change appeared to drive their engagement with this intervention.

#### 3.3.2 Theme 2: the difficult journey through DBT

This theme, comprising three sub-themes, describes the content and processes of DBT that were reported to affect psychological recovery, thereby exploring how practical aspects, such as mode of delivery, were perceived as helping or hindering progress.

##### 3.3.2.1 Sub-theme 2.1: from theory to practice

The content of DBT, particularly the structured worksheets and skill-building resources, was described as integral to navigating the journey through DBT. Skills and content from all four modules (*mindfulness, interpersonal effectiveness, distress tolerance* and *emotion regulation*) helped individuals to better cope with distress and challenges in their daily lives, through improved impulse control, self-regulation and self-awareness. Module content enabled individuals to learn how to pause and process thoughts and feelings with greater clarity, enabling different behavioural responses to be selected.

Particularly helpful was having a variety of skills to choose from (“*I can get myself into wise mind and use the skills. And then if I need to blow off steam, I can just go through the skills and pick out the ones to calm myself down”*, Cunningham et al. (85), p.2005) and facilitators giving relatable examples (*“Role play [ … ] you know things that we would have to cope with. Like say we are in a supermarket or something”* Roscoe et al. (78), p.271). These factors allowed application of learned content more broadly in participants' lives, supporting skill use in various situations. As a result, skills were practiced more often and became second nature:

“Since the beginning what he taught us was what to do in situations like that, so I would always try to put it to the test, I even got the point where I could do it without thinking” (Pardo et al. (101), p.6).

Furthermore, the tangible and practical nature of DBT skills, and how these were learnt contributed to psychological change. The learning process including the use of physical items (such as sensory boxes and diary cards) and practical strategies (such as grounding or mindfulness techniques) helped individuals to better process and apply the new knowledge to their lives. Participants elaborated on how structured resources were useful, because they helped track progress and maintain motivation because people could visually see their progress week by week:

“…when it first started, I used to have quite high scores on my diary card, mmm … and now I only have like ones and twos…!” (Roscoe et al. (78), p.271).

Participants also reported that information being taught in a way that felt less abstract and more accessible contributed to psychological recovery. However, some aspects were experienced as less helpful. Participants found the DBT terminology complex (“*like a lot of the jargon that’s read out to you – does that come with subtitles?”*, McSherry et al. (81), p.2) or the therapeutic concepts too abstract (*“um I didn’t like the mindfulness bit when they said you were hot headed and in a hot mind or a cool mind. I didn’t understand that very well”*, Crossland-Hewitt (72), p.212).

##### 3.3.2.2 Sub-theme 2.2: transformative relationships - self and others

While undertaking DBT, perhaps unsurprisingly, relational factors played a crucial role in the therapeutic process, including peer support which emerged as a significant influence, with group members learning from one another’s experiences, as intended by Linehan (1, 2, 5):

“…so, we give each other tips and exchange ideas and sort of: ‘I liked this, it might work for you too” (Ohlis et al. (53), p. 418).

While DBT structure varied across studies, common core components, such as crisis support and the support of the group, were important because these components were reported to reduce feelings of isolation, which helped maintain engagement.

Participant narratives also showed that validation from therapists was highly valued by participants, especially given their trauma histories, because this validation allowed them to feel accepted for who they were. However, importantly, therapeutic relationships that provided both psychological safety and challenge were pivotal in fostering behavioural change, because these relationships they facilitated growth and challenged individuals with a history of avoidance and interpersonal conflict, to make behavioural changes in a relatively comfortable way:

“My therapist is fantastic because he doesn’t sugar-coat anything, he tells me how it is [ … ] if I don’t want to come to therapy, he helps me understand where that feeling is coming from and to evaluate the impact that not coming would have” (Barnicot et al. (61), p.217).

Participant narratives indicated that DBT had led to a dramatic shift in their perspective, with their view of themselves, others and the world changing through the DBT experience. Attitudes shifted towards greater self-acceptance, which often led to improved symptoms. An increased sense of self-confidence, purpose and hope for the future was reported, which contributed to psychological recovery and long-term maintenance of gains:

“It’s thanks to DBT that I’m alive today … now I can see myself as a person that shall go on living … I don’t think I’d be around anymore, if it wasn’t for DBT…” (Perseius et al. (86), p222).

##### 3.3.2.3 Sub-theme 2.3: scaffolding and supporting change

Additionally, the structure and format of DBT, which are essential elements of the broader therapeutic journey, played a significant role in shaping participants’ progress.

Groups were described as engaging *(“some balloons in the air and also when we watched these short [film] clips. I think that’s fun.”*, Meyer et al. (97), p.674). Individual therapy was described as intense and difficult, meaning that the more entertaining approach taken within group delivery was experienced as helping to prevent individuals falling into their previous patterns of isolation, avoidance and overwhelm:

“To feel as though you are not alone in the world with your problems [ … ] I feel very isolated in the community [ … ] DBT helped me to feel part of something” (Russell & Siesmaa (75), p.52).

Concerns about confidentiality and the prevention of discussing or utilising self-harm commonly being a group rule were described as challenging aspects, because some individuals described feeling unsafe, and pre-existing feelings of uncertainty and fear were reinforced:

“Even now that frustrates me, cos I think, what if I’m not allowed to self-harm, what do I do … it’s not an option anymore” (Hodgetts et al. (84), p.175).

Polarised views regarding online delivery reflected individual circumstances. For some, online delivery promoted attendance and was helpful *(“I thought it was very good that it was online based, because I did not have to keep any appointments*”, Vasiljevic et al. (50), p.60), meaning their therapeutic exposure, and therefore possibly their therapeutic gains, increased. Others described how online delivery facilitated disengagement (“*was less willing to work on things over a video call, whereas when we had face-to-face sessions, I was always more enthusiastic and ready to talk and work”* (Ramzan et al. (95), p.8). Importantly, views regarding delivery format highlighted the increased gains and engagement when intervention aligned with patient preferences.

Together, the content, relational processes, and delivery format of DBT each played a crucial role in participants’ engagement with the intervention. While each element presented unique obstacles, they ultimately contributed to the transformative process of learning new coping strategies and gaining better control over emotional responses.

#### 3.3.3 Theme 3: patients’ path for the future

This theme emerged through people reflecting on their DBT intervention ending and it consists of two sub-themes: *therapeutic gains* and *future suggestions for therapy.* These sub-themes capture the perceived transformative impact of DBT and offer ideas for future intervention refinement.

##### 3.3.3.1 Sub-theme 3.1: therapeutic gains

As individuals reflected on their experiences of DBT, the transformative impact of the intervention became apparent. Individuals reported numerous relational gains, including fewer conflicts with others and improved parenting skills (*“I’m more assertive with the way that I* sp*eak to people and so people aren’t treating me the way they were and I’m not bottling it in and getting angry at them”*, Francis et al. (47), p.1255). Often these relational gains stemmed from personal gains, such as decreased impulsivity, increased emotion regulation ability, the development of adaptive coping skills, or improved self-esteem and compassion, leading to greater prioritisation of their own needs:

“Something I wouldn’t have done before DBT, I am thinking of myself and what’s best for me and my whole family … The whole time gently pulling back because I think it will be healthier for me” (Gillespie et al. (57), p.9).

An increased sense of self-kindness was experienced by many, with individuals learning to show themselves greater compassion when they were struggling. This compassion often coincided with better boundaries, and subsequently less emotional distress:

“I have come to realise I do not judge myself in the same way as everyone else does. I’ve been so super hard on myself. I recognise now that I have to judge myself the same way I judge others and be kind to others” (Meyer et al. (97), p.675).

New skills had incredible value in people’s lives, which was evident across the reviewed studies, with some people reflecting how they would not want to lose them in future. Additionally, some studies considered the long-term impact of therapy, with the value still evident years later, while the importance of skill maintenance was reflected on to maintain therapeutic gain:

“One thing I’ve learned is you just gotta keep doing them. Like, if you don’t keep up this, it’s like learning a language, if you don’t keep it up, you lose it” (McColl et al. (43), p. 8).

##### 3.3.3.2 Sub-theme 3.2: future directions

While participants expressed overall satisfaction with their experiences of DBT, several shared suggestions to enhance the therapeutic journey for future participants. For example, some reported that their programme should have been longer, or offered refresher sessions, to help embed the skills more.

Several participants expressed that simplifying language and using more relatable concepts was needed, given it would prevent people feeling inferior and reduce attrition, particularly for those who had previously struggled with traditional therapy approaches:

“I was ready to quit because I couldn’t get these big words, and I didn’t know some of the language” (Cunningham et al. (85), p.253).

Some participants reported feeling that broader representation within therapists would be beneficial. If group members had seen their characteristics better reflected in group facilitators, they expressed that it might have increased therapeutic gain given that therapeutic relationships were a process that supported change:

“I found that a lot of the [therapists] are like cis-gendered or heterosexual. So maybe if there was some … queer or … transgender or non-binary [therapists] in DBT” (Camp et al. (88), p.13).

Overall, this theme captured participants’ reflections on the lasting impact of DBT, including personal growth, improved relationships and increased self-compassion. It also considered refinements for future intervention delivery to enhance these impacts further.

## 4 Discussion

This systematic review synthesised data from 32 studies to identify key themes relating to patient experiences of DBT, across mental health conditions and age groups. It considered the processes (peer support, validation, psychological safety, changed perspectives, increased sense of purpose and hope) patients perceived as contributing to, or impeding, therapeutic change, as well as considering ways to enhance DBT offers to maximise patient gains in the future. The findings highlight the significance of both pre-treatment (trauma, self-awareness, coping behaviours and previous therapy) and in-treatment (structured resources, modular content, practical skills and relatable examples) patient experiences in shaping engagement and reported psychological outcomes, emphasising how these specific aspects of DBT content and delivery can either promote or hinder progress transdiagnostically.

Findings from previous reviews of DBT (e.g., (26, 27)) highlighted the importance of skill development, hope, and the therapeutic relationship. The current review builds on this identified importance by offering a deeper understanding of the experiential aspects of DBT that contribute to transdiagnostic change, such as the development of self-compassion, sustained motivation, and peer connection, across both adult and adolescent populations. The current review findings offer a unique contribution to clinical psychology and can be operationalised through trauma-informed clinical training, experiential learning, supervision that emphasises therapist relatability, and service development that supports diverse, accessible, and modular DBT delivery.

Relational factors, such as feeling safe, validated, and connected to both peers and therapists, have been recognised as contributing to therapeutic gains in various group therapies (103, 104), a sentiment supported by the current review. However, the current review also found that certain DBT components, such as crisis support, bolstered these feelings of safety and connection, ultimately amplifying reported therapeutic outcomes for some individuals. Furthermore, given the transdiagnostic experiences of trauma in the 32 reviewed studies, feelings of low self-esteem and fear were typically prevalent for those undertaking DBT. For these participants, experiencing validation and safety in group therapy may be particularly impactful compared to other clinical populations (105).

The current review highlights the value of skills across all four DBT group components, with patients often benefiting from the variety offered, underscoring the strengths of DBT’s modular design (5, 48). Notably, participants frequently reflected on using these skills in everyday life. This focus on practical skill application contrasts with previous research, which often considers DBT’s impact through outcomes like reduced self-injury or mood instability (17, 106, 107). As DBT’s treatment hierarchy, understandably, prioritises self-injury and suicidality, these measures have traditionally dominated research. However, with DBT’s growing use among more diverse clinical groups, in which such behaviours may be less common, there is a need to expand outcome measures. The current review suggests that in these cases, exploration of real-world skill use and emotion regulation through interview may better capture DBT’s therapeutic impact.

Variation in DBT formats across the included studies, such as stand-alone skills training groups versus full DBT programmes, may have influenced the review’s findings. These differences in treatment intensity, structure, and therapeutic components (e.g., absence of individual therapy or phone coaching in skills-only formats) could have shaped how participants experienced the intervention and the specific mechanisms of change they reported. As a result, some themes identified in the synthesis might reflect format-specific experiences rather than transferable features of DBT as a whole.

### 4.1 Clinical implications

Clinical recommendations, developed based on the identified themes and grounded in qualitative data, are provided to inform the delivery of DBT and guide practice (see **Table 6** for details). As this review highlights the importance of aligning intervention offers with patient preferences, and the variation in patient preferences (e.g., regarding favoured delivery and modules), commissioners and clinicians could consider adapting these elements based on patient feedback and service need, as recommended in **Table 6**. At a broader level, these findings may also inform public policy by supporting investment in accessible, modular, and patient-centred DBT programmes that foster self-compassion, sustained motivation, and peer connection across diverse populations, thereby shaping equitable mental health service provision and guiding resource allocation at a system’s level.

**Table 6:** Suggested clinical recommendations for DBT interventions with skills groups.

Therapy phase	Recommendations
Patient Engagement	Trauma screening to be completed pre-intervention to ensure suitable participants who may benefit from DBT are identified. This would provide an opportunity to discuss the trauma’s impact on functioning and can scaffold conversations regarding how such experiences can be used as motivation to engage, as well as how the intervention can help reduce unwanted consequences of these experiences. Facilitating patient choice by services offering multiple delivery options (when feasible), such as both online and in-person DBT groups, to enhance therapeutic outcomes. Clinicians should consider the underlying beliefs when assessing emotional dysregulation difficulties. This will help in evaluating the suitability of DBT versus other therapies, like Compassion-Focused Therapy, and hopefully increase engagement when the model better aligns with patient needs.
Retention	Adaptations to session language and resources should be considered, simplifying terminology and abstract concepts to better suit the needs of the target patient group. Clinicians to be mindful that when patient groups have high levels of relational trauma, and a higher need for containment, crisis support should be offered alongside DBT skills intervention. DBT skill interventions offered should include the four recognised modules (mindfulness, interpersonal effectiveness, distress tolerance and emotion regulation) as outlined by Linehan (5, 48) because content from all four appear valuable to participants. Clinicians to remain mindful that therapeutic relationship is consistently reported as a contributor to continued attendance and therapeutic gain. Clinicians should be mindful of the intersectionality of each patient’s identity within the intervention and ensure group content and examples are inclusive.
Other	If feasible, it may be helpful to offer additional ‘top up’ sessions for participants to help sustain and build on therapeutic gains. When evaluating group DBT interventions, clinicians should consider some measure of practical skill application, given that patients often describe everyday skill application as a significant and positive intervention result.

### 4.2 Strengths, limitations and future research

This review involved a comprehensive and systematic search, with data synthesised from 32 studies, reflecting the voices of 414 people, from ten countries, spanning 21 years. This review deliberately included diverse voices across age, sex, and clinical groups in an attempt to provide a holistic view of experiences. Furthermore, 31 of the 32 included studies were rated as having high methodological quality on the CASP. The inclusion of a second independent reviewer further supported the review’s strong methodological rigour (29). However, the lack of methodology exclusion criteria enabled the inclusion of a lower quality study (82), which future studies may seek to exclude.

The CASP tool is widely used and was chosen to enhance methodological rigour and credibility, ensuring the synthesis was based on high-quality evidence. However, subjectivity and quality concerns regarding the CASP are acknowledged, for example, its lack of sensitivity to validity when compared with other tools (108). Furthermore, although selected for their relevance to the topic area, the authors acknowledge possible limitations associated with the databases used; for example, that they might have over-prepresented research from high income countries leading to a skewed sample. In addition, this review did not incorporate trial registries (e.g., CENTRAL, ICTRP, ClinicalTrials.gov) or grey literature sources such as dissertation repositories. Including these might have yielded a more comprehensive dataset and ensured broader coverage of the existing evidence base. While we opted for the inclusion of peer reviewed academic literature only, a future review may consider the inclusion of grey literature.

The included studies varied substantially in intervention length (ranging from three to 36 months), delivery model (online vs. in-person), and treatment structure (e.g., stand-alone skills groups vs. full DBT programmes). This heterogeneity in intervention design and implementation might have contributed to variability in outcomes, complicating the synthesis and interpretation of reported effectiveness.

Ethnicity was reported in only 12 of the 32 studies included in the systematic review, suggesting a significant gap in the representation and reporting of participant diversity. This lack of demographic detail raises concerns about the potential homogeneity of study populations and might limit the transferability of findings across ethnically diverse groups. These omissions underscore the importance of applying an intersectional lens in research and highlight the need for more inclusive clinical care practices that account for the complex ways in which ethnicity, culture, and other identity factors shape health outcomes.

As non-English studies were excluded due to time and resource constraints, potentially introducing publication and language biases, future reviews may want to include studies published in other languages. Nevertheless, included studies spanned ten countries, six of which do not have English as their primary language.

This review also synthesised various intervention formats that included a group element, including traditional and skills group only DBT. Future studies may wish to select one particular variation to focus on to explore if there are any unique benefits or challenges associated with each, offering a deeper understanding of their relative value.

### 4.3 Conclusion

To the authors’ knowledge, this is the first review to comprehensively synthesise qualitative research on patient experiences of DBT, including a group component, across mental health conditions and clinical populations, as well as age groups from adolescence to adulthood. The findings supported the previously identified multifaceted impact of DBT on adolescents’ and adults’ emotional and relational well-being transdiagnostically. These findings build on previous reviews by highlighting DBT’s transdiagnostic impact on self-compassion, motivation, and real-world skill application. Relational elements, such as feeling validated and psychologically safe, were particularly important for individuals with trauma histories, reinforcing the value of DBT’s structure and delivery. Furthermore, given patients' complexity offering DBT in flexible formats is beneficial to improve accessibility. Notably, participants described everyday use of skills as a key therapeutic outcome, suggesting future research should include measures of functional change, not just symptom reduction. Clinical recommendations are provided to guide more responsive and inclusive DBT delivery, promoting long-term therapeutic benefit and enhancing DBT’s ability in addressing the diverse needs of patients across different backgrounds and settings.

## Data Availability

Publicly available datasets were analysed in this study. All data can be found in tables in the main text.
